# Developing a new protocol for monitoring vegetation in limestone pavement

**DOI:** 10.1038/s41598-025-05931-z

**Published:** 2025-07-01

**Authors:** Carly Stevens

**Affiliations:** Lancaster Environment Centre, Lancaster University, Lancaster, LA1 4YQ UK

**Keywords:** Alvar, Habitat management, Karren, Karst, Lapaiz, Monitoring, Ecology, Ecology

## Abstract

Limestone pavements are a rocky habitat formed of slabs of rock with crevices (grikes) between them where a rich community of plants can grow. The habitat is important for conservation but there is currently a lack of evidence to support their management and conservation. Putting monitoring in place when management changes are made will help to build an evidence base to support decision making in conservation but it can be challenging to monitor vegetation in limestone pavements. Here I present a monitoring protocol suitable for use by conservation practitioners and others working in limestone pavements. Following discussion with practitioners, 24 pavements were selected to represent the variety of pavements in Great Britain. Transect surveys were conducted to identify the optimal length of grike for survey. Based on existing practice in other habitats, a suitable approach to when management changes are being made would be to use a before-after-control-impact design. Within each 2500 m^2^ plot twenty 10 m transects, randomly placed in grikes, should provide sufficient coverage for repeat monitoring. Species should be identified to a species level wherever possible and cover estimated. The results of the analysis support the view that the new method is a reliable approach.

## Introduction

In order to manage habitats optimally we need a good understanding of the vegetation found within them. Accurate and repeatable surveys are needed to monitor change in response to global change drivers, to establish a baseline and monitor the success or otherwise of management approaches and habitat condition assessment^1^. However, monitoring in limestone pavements presents a number of practical challenges.

Limestone pavements are rocky habitats that were formed during glaciation. The habitat we see today consists of a rocky surface with slabs of rock called clints, interspersed with fissures called grikes. The clints can range in size from a few centimetres to several metres wide, while grikes are typically from around a couple of centimetres wide up to a metre and can be several metres deep. A majority of the vegetation in limestone pavements is found growing within the grikes. The vegetation of limestone pavements can be very variable depending on tree cover and pavements are often classed as either open or wooded^2^. Ferns can be abundant together with herbaceous species typical of woodland commonly occurring. Limestone pavements support a number of plant species that are rare in Great Britain including northern holly-fern (*Polystichum lonchitis*), dark red helleborine (*Epipactis atrorubens*) and baneberry (*Actaea spicata*). Limestone pavement vary considerably in their physical structure which has important implications for the vegetation found in them^3^.

Limestone pavements are found in a number of different parts of Europe and Canada with largest areas found in Austria and Ireland^4^. In Great Britain limestone pavements are mostly found in the north of England with the largest areas found in Yorkshire, Lancashire and Cumbria together with smaller areas in Wales and Scotland, covering a total area of approximately 28 km^2^^4^. Limestone pavements are glacial features, formed by the movement of ice sheets during the last glaciation. This exposed areas of limestone which have since been eroded by rainfall to create the grike and clint structure. Pavements usually occur as part of a matrix of habitats including calcareous grassland, woodland, heathland and bog. Pavements in the Great Britain are a mix of wooded and open pavements with Pigott and Pigott^5,6^ suggesting that some have been open since the iron age. Open pavements are often managed by grazing with either cattle or sheep but further research is needed to identify optimal management approaches^7^. Limestone pavements are an irreplaceable habitat and are protected in the UK through Limestone Pavement Protection Orders^8^ and many individual sites are protected as National Nature Reserves or Sites of Special Scientific Interest^8^.

In other habitats vegetation is often sampled using quadrats but in limestone pavements inappropriate placement or size of quadrat, or an inappropriate sampling design could result in zero grikes to survey or multiple grikes. The use of transects along grikes provides a suitable alternative but can again be fraught with difficulties if the pavement does not have a regular structure or grikes are shallow and open.

As with many disciplines, botanical and other ecological surveys suffer from a lack of repeatability^9,10^. Providing a rigorous, tested protocol for surveyors to follow is critical when trying to identify changes to a site over time^11^ and can improve repeatability. In their extensive survey of British pavements Ward and Evans walked over pavements producing a species list with abundance on a scale of 1 to 3^12^. This approach provides a rapid assessment but is scale dependent and somewhat subjective. In repeating this survey Stevens^7^ found that there were likely sources of error in identifying exact location of some surveyed pavements, estimation of species cover and surveyor effort. In 1982 Silvertown^13^ recommended a monitoring approach that sampled independent grikes. Silvertown’s approach involved measuring the lengths of between 50 and 100 grikes and using this to create a frequency histogram. The histogram was then used to determine the length at which 95% of grikes were included. Transects were set up at right angles to the grikes being investigated and the length identified in the previous step was used as a minimum separation distance for the transects. This meant that grikes had a 95% chance of being independent and avoided double counting of species that were spreading by rhizomes. Silvertown recommended that samples could be taken at regular intervals along the transect (e.g. every second grike) to investigate species distributions or in every grike to assess species interactions. While this method is thorough and repeatable it is very time consuming making it less suitable for application in less academic studies. While the approach works very effectively in pavements with a regular structure and large clints, it is less practical in pavements with a very open or irregular structure, damaged pavement, patches of pavement within other habitats or small areas of pavement.

This manuscript aims to describe and evaluate a rapid but repeatable approach to monitoring limestone pavement vegetation which can be applied in all pavements. This is particularly needed when monitoring response to management changes, something which currently undertaken quite rarely but which could provide a wealth of information for conservation practitioners around the most appropriate management approaches.

## Methods

While compiling the data for a national study of limestone pavements^7^ virtually all limestone pavements in the Great Britain were visited. Having such a clear overview of British limestone pavements provided a strong understanding of the diversity of pavements found in Great Britain. This was complemented by discussions with conservation professionals working in limestone pavements to understand the extent and nature of monitoring that was already conducted, ideas for how monitoring should be conducted and the barriers to conducting monitoring.

Twenty-four individual pavements were selected to represent the variety of structures, extent and vegetation types that were observed in Stevens^7^. These pavements were visited between June and September 2024. At each site a 2500 m^2^ area as either 50 × 50 m or 25 × 100 m depending on the shape of the pavement was delineated. The area was selected to be representative of the pavement and to contain as much pavement as possible (though it was not necessary for the whole area to be pavement as areas of pavements can be very irregular and patchy). Within this area 10 m transects were taken. Transects were located randomly within grikes or along the edges of clints and did not need to be in a straight line as long as they stayed within a grike or along a rock edge where the first 10 cm perpendicular to the transect was recorded. Twenty-five transects were collected within each site taking care to ensure that the same area was not surveyed twice. 250 m of transects were recorded in all of the pavements surveyed, in some this represented almost all of the grikes present whereas others it was not the case. Species present in each transect were recorded.

The software Estimate S^14^ was used to calculate Coleman rarefaction curves^15,16^ for sample-based frequency data providing an expected species richness for a sub-sample of the pooled total species richness, based on an empirical reference sample (S) and a species richness estimate for incidence-based data Chao 2^17,18^, was used to determine the most appropriate total transect length.

## Results and discussion

Monitoring of vegetation change when management changes are made in limestone pavements has the potential to provide an evidence base for the management of limestone pavements which is currently lacking in Great Britain. In developing a monitoring protocol compromises need to be made between describing the habitat accurately and the time required to conduct the survey.

### Sampling area

The 2500 m^2^ sampling area was selected to be a large enough area to be representative of pavement vegetation and structure in most pavements and to minimise the impact of small errors in relocating plots, while being small enough to allow relatively rapid sampling. This sampling area was selected based on discussion with a range of practitioners. Marking plots in limestone pavements can be challenging, in most habitats ecologists typically mark plot corners using either corner stakes or buried metal markers which can be relocated with a metal detector. Neither of these approaches is possible in such a rocky habitat. In the UK it is illegal to damage the rocks in limestone pavement^8^ and so special permission must be obtained to mark the rock directly, for example with paint. Pavements are also commonly publicly accessible and grazed, meaning markers could be moved or removed. Consequently, there is often a need to record plot locations with GPS. While differential GPS can provide highly accurate locations such equipment is frequently not available to conservation practitioners and hand-held GPS must be used. These typically have an error of several metres necessitating the need to minimise the impact of these errors when relocating monitoring plots.

The sampling area should be dominated by pavement for this monitoring protocol to be effective, and should be placed in a location that is representative of the wider pavement. However, if plots can be permanently marked or the area of pavement is small, smaller areas could be used. If a pavement is particularly large or variable it might be necessary to use more than one survey area.

### Sampling approach

While the ideal approach to assessing the response of a habitat to human-induced perturbations such as a change in management is a fully replicated and controlled experiment, a Before-After-Control-Impact (BACI) approach with less replication can provide an effective alternative^19^. BACI has been widely used in a range of different situation to assess population changes following perturbation e.g^20,21^. Although the approach has been criticised, it offers the advantage that it can be applied to non-random site^19^ so is appropriate to apply where targeted management activities are being conducted. One of the major criticisms of BACI is that there is commonly only one unreplicated impacted site^22^ and a lack of replication in controls, consequently when using BACI it is recommended that at least two control plots are used^23^. Control plots need to be similar to the monitored area. In limestone pavements this would mean a pavement of a similar structure, with similar vegetation and management (before any changes are made). It can be very difficult to identify suitable control plots and this approach multiplies the effort required for monitoring which may be prohibitive, as such it is not always possible to introduce control plots. Using a simple before and after approach is less accurate and means that changes that occur in the wider environment (e.g. high rainfall in a given year) cannot be separated from the effects of the management change made^24^. Ideally data would be formally analysed formally to examine changes in abundance of key species, species richness and diversity with ordination approaches to show changes in composition but this isn’t always possible outside academia. However, even basic analysis showing changes in average abundance of key species and species richness provides evidence of change. This data can also be used to assess change in conservation objectives such as increases in indicator species such as the positive and negative indicator species identified in the UK Common Standards Monitoring guidance^2^ rare species or invasive species.

### Sampling design

Sampling takes place in grikes since this is where pavement species are typically found. For this protocol grikes are broadly defined as areas between rocks (with no specific width to depth ratio) and areas around the edges of rocks where the pavement becomes open have also been included (only first 10 cm) to allow suitable lengths of continuous recording when pavements have an open structure. Samples should be located at random, something which can be achieved using random numbers and pacing out locations then recording the nearest grike. However, depending on the focus of the monitoring it may be necessary to supplement this information with notes on extent of emergent or clint top vegetation, or signs of browsing, especially if grazing is a focus of the study.

**Fig. 1:**
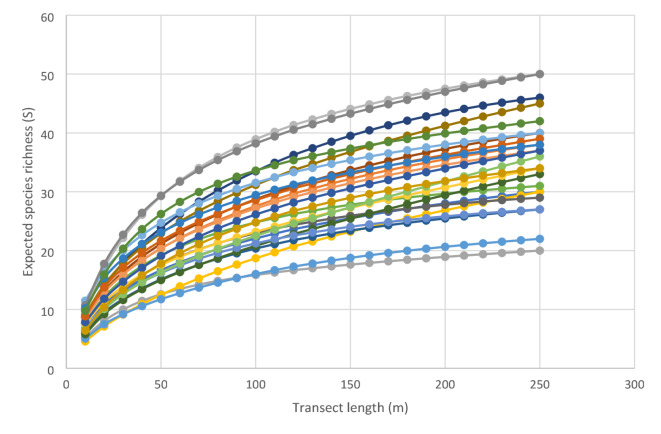
Expected species richness (S) for a given length of transect for 2500 m^2^ areas within 24 limestone pavements (represented by different colours).

**Fig. 2:**
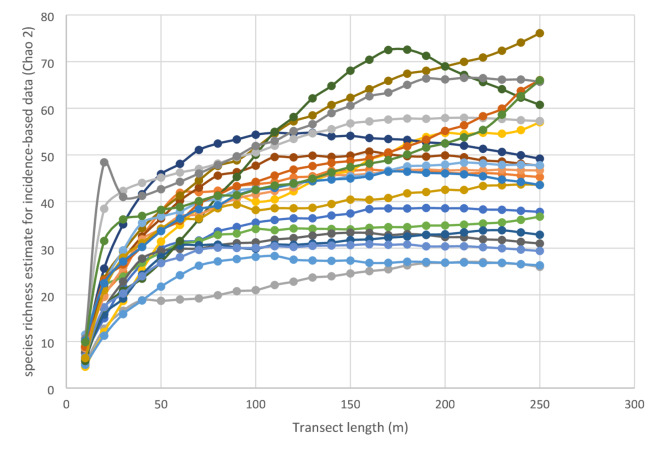
Species richness estimate for incidence-based data (Chao 2) for a given length of transect for 2500 m^2^ areas within 24 limestone pavements (represented by different colours).

An ideal approach would be to survey all vegetation but this is very time consuming. Fixed transects would offer a highly repeatable approach but the difficulties with marking plots apply equally to making transects. Therefore, surveying random grikes to a sufficient level that a majority of species are captured provides an alternative. Surveying 250 m of grike in the form of 20 randomly located 10 m transects resulted in species accumulation curves which either plateaued or were close to doing so (Fig. 1). In some pavements almost all of the grikes were surveyed with 250 m and so in these cases although the curve appears not to have plateaued further sampling could not be conducted. Utilising the equations for the relationships between species richness and transect length showed that surveying 200 m of grike accounted for more than 80% of expected species richness at 250 m in all pavements and 90% or more of expected species richness in all but two pavements. It also represented a level at which species richness estimated by Chao 2 was at a maximum for all but three pavements (Fig. 2). This means that exact relocation of grikes is not necessary since a high proportion of total species richness is captured, even in a species rich pavement.

Along each transect species should be identified to a species level wherever possible. This is likely to require good identification skills (equivalent to BSBI FISC level 4 or possibly 5)^25^. Not recording to a species level may result in a loss of sensitivity as species composition changes. It also limits the use of data with ecological indices such as Ellenberg values^26^ which may help interpret vegetation change. However, recording difficult groups to a genus level will still provide data that can be used to assess change and so this should not be a reason for not monitoring.

Incorporating a measure of abundance means that monitoring is sensitive to changes in abundance. The population of an individual species, including one of conservation interest, could have decline sharply but this would not be recorded until it is eliminated from the site entirely if only presence and absence is considered. Counting frequency is very time consuming, especially with species such as *Mercularis perennis* (dog’s mercury) which grow in dense clumps. It is also likely to result in errors as abundance will vary according to grike width leading to a lack of repeatability. Using Domin values^27^ to estimate abundance is somewhat subjective^28^ but provides a compromise between a challenging and time-consuming approach of directly counting frequency and a rapid assessment. Percentage cover is even more subjective and not easy to assess accurately over 10 m length so using Domin values reduces subjectivity. Comparisons of Domin data should be made using the median and range since Domin scores are not in even categories.

## Conclusion

Implementing monitoring when management changes are made is an important part of generating evidence to decision making. While limestone pavements are a challenging habitat to monitor there are options that have the potential to produce accurate and reliable data. In limestone pavements a suitable approach would be to use a BACI design with 2500 m^2^ plots. Within each plot twenty 10 m transects randomly placed in grikes should provide sufficient coverage for repeat monitoring. Species should be identified to a species level wherever possible and cover estimated on a Domin scale. Evaluation of the data collected from 24 sites supports the view that the new method is a reliable approach. While a survey based on grikes is specific to limestone pavements there are lessons that can be carried over to other rocky habitats.

## Data Availability

Data available from the Lancaster University repository Pure: 10.17635/lancaster/researchdata/695 or by contacting Carly Stevens c.stevens@lancaster.ac.uk.
